# Hemozoin (Malarial Pigment) Directly Promotes Apoptosis of Erythroid Precursors

**DOI:** 10.1371/journal.pone.0008446

**Published:** 2009-12-24

**Authors:** Abigail A. Lamikanra, Michel Theron, Taco W. A. Kooij, David J. Roberts

**Affiliations:** 1 Nuffield Department of Clinical Laboratory Sciences, John Radcliffe Hospital, Oxford, United Kingdom; 2 National Blood Service Oxford Centre, John Radcliffe Hospital, Oxford, United Kingdom; 3 Wellcome Trust Genome Centre, Wellcome Trust Sanger Institute, Cambridge, United Kingdom; 4 Department of Parasitology, Max Planck Institute for Infection Biology, Berlin, Germany; Health Canada, Canada

## Abstract

Severe malarial anemia is the most common syndrome of severe malaria in endemic areas. The pathophysiology of chronic malaria is characterised by a striking degree of abnormal development of erythroid precursors (dyserythropoiesis) and an inadequate erythropoietic response in spite of elevated levels of erythropoietin. The cause of dyserythropoiesis is unclear although it has been suggested that bone-marrow macrophages release cytokines, chemokines or lipo-peroxides after exposure to hemozoin, a crystalloid form of undigested heme moieties from malarial infected erythrocytes, and so inhibit erythropoiesis. However, we have previously shown that hemozoin may directly inhibit erythroid development *in vitro* and the levels of hemozoin in plasma from patients with malarial anemia and hemozoin within the bone marrow was associated with reduced reticulocyte response. We hypothesized that macrophages may reduce, not enhance, the inhibitory effect of hemozoin on erythropoiesis. In an *in vitro* model of erythropoiesis, we now show that inhibition of erythroid cell development by hemozoin isolated from *P. falciparum* is characterised by delayed expression of the erythroid markers and increased apoptosis of progenitor cells. Crucially, macrophages appear to protect erythroid cells from hemozoin, consistent with a direct contribution of hemozoin to the depression of reticulocyte output from the bone marrow in children with malarial anemia. Moreover, hemozoin isolated from *P. falciparum in vitro* inhibits erythroid development independently of inflammatory mediators by inducing apoptotic pathways that not only involve activation of caspase 8 and cleavage of caspase 3 but also loss of mitochondrial potential. Taken together these data are consistent with a direct effect of hemozoin in inducing apoptosis in developing erythroid cells in malarial anemia. Accumulation of hemozoin in the bone marrow could therefore result in inadequate reticulocytosis in children that have adequate levels of circulating erythropoietin.

## Introduction

Severe malaria caused by *P. falciparum* causes many different syndromes which culminate in more than a million childhood deaths each year. In young infants in holo-endemic regions of Africa the predominant syndrome of severe malaria is severe malarial anemia (SMA) (reviewed in [1], [2]). SMA is due not only to increased hemolysis of infected and non-infected red blood cells (iRBC) but also due to a striking degree of abnormal development of erythroid precursors in acute and in chronic infection [3], [4] and an inadequate erythropoietic response in spite of elevated levels of erythropoietin (Epo) [4], [5], [6]. The distribution of erythroid precursors in the cell-cycle is also abnormal with an increased number of cells in the G_2_ phase compared with normal controls [7], [8]. In simian and murine models of malaria, ineffective erythropoiesis also contributes to anemia [9], [10], [11].

The pathology of inadequate erythropoietic responses associated with malaria infection has not been established. The systemic pro-inflammatory cytokines tumor necrosis factor-alpha (TNF-α) and interferon-gamma (IFNγ) have been associated with SMA [12], [13] and reviewed by McDevitt *et al* [14]. However other experimental and clinical studies suggest that by-products from the asexual stage of infection such as the ring surface protein 2 (RSP-2) [15], [16], [17], glycophosphatidylinositol (GPI) anchors of merozoite proteins [18], [19] and hemozoin (or its synthetic analogue β hematin) [4], [20], [21] also contribute to the pathology of SMA.

Hemozoin is formed in the food vacuole of developing intra-erythrocytic parasites, as toxic heme remaining after digestion of hemoglobin forms a crystalline dimer of α hematin, complexed with lipid and protein. Hemozoin crystals closely resemble β hematin, consisting of a ferric ion within a protoporphyrin IX ring structure [22]. Hemozoin released after the lysis of iRBC is more heterogeneous and is phagocytosed by the reticulo-endothelial system, where it is readily observed within macrophages of the bone marrow and spleen [23] and reviewed in [24].

Hemozoin and its constituents affect the function of host cells. Schwarzer, Arese and colleagues showed inhibition of macrophage function by hemozoin including reduced production of the pro-inflammatory cytokines IL-1β and TNF-α and reduced phagocytic activity and oxidative burst [25], [26]. Others have shown that hemozoin induces production of the same inflammatory cytokines [27] and that synthetic hemozoin enhances IFN-γ-inducible nitric oxide synthase (iNOS) and the chemokines macrophage-inflammatory protein (MIP)-1α, MIP-1β, MIP-2, and monocyte chemo-attractant protein-1 (MCP-1), that may mediate enhanced migration of macrophages and neutrophils [28], [29]. These cytokines and chemokines have been shown to inhibit erythropoiesis [30], [31], [32], [33], [34]. More recently synthetic hemozoin administered to mice has been shown to induce IL-6 production via the release of uric acid [35].

The pro-inflammatory effects of hemozoin have been attributed to the oxidative properties of heme. The ferric ion co-ordinated in the heme moiety is a potent catalyst of free radical production through the Fenton reaction (for review see [36]). Experimental studies of the effect of hemozoin on monocytes have shown that abnormal monocyte function was associated with the mono-hydroxy derivatives of the poly-unsaturated fatty acids (OH-PUFAs) in hemozoin produced after metabolism of hemoglobin by the parasite [37]. The biologically active lipo-peroxides, such as 15-(s)-hydroxyeicosatetraenoic acid (15-S-HETE) and 4-Hydroxy-2-Nonenal (4-HNE) are potentially inhibitory to the growth of erythroid cells [38]. Furthermore, 15-S-HETE (a mono-hydroxy derivative of arachidonic acid) was shown to inhibit differentiation and maturation of dendritic cells [39]. Taken together, these data have supported the hypothesis that during malaria infection, bone marrow macrophages contribute to the inhibition of erythropoiesis indirectly or directly by oxidative stress.

Previously we have shown that hemozoin may inhibit erythroid precursors *in vitro* at concentrations found in the peripheral blood of children presenting with anemia and malaria [4]. The mechanism of this inhibition has not been established. Inhibition of erythropoiesis may result from disturbing the balance of anti-apoptotic and pro-apoptotic factors that are required for normal erythroid cell development. In the later stages of this development, cell death has been shown to result from increased activation of caspases, withdrawal of Epo or stimulation of the death receptors Fas (CD95) or TRAIL (for review see [40]). Increased apoptosis of developing erythroid cells has also been observed in a variety of malignant, genetic and inflammatory disorders including myelodysplasia [41], myeloma [42], rheumatoid arthritis [43], septic shock [44] and thalassaemia [45].

To understand the mechanism of inadequate erythropoiesis in more detail during malaria infection we used a two phase liquid culture system of erythropoiesis that contains macrophages [46]. We show that macrophages protect erythroid cells from hemozoin and that reduced erythroid expansion was accompanied by increased activity within the extrinsic and intrinsic pathways of apoptosis. These events occurred in the absence of inflammatory mediators and macrophages suggesting that accumulation of hemozoin in the bone marrow could contribute to the severity of anemia in children with chronic malarial infection.

## Results

### Inhibition of Erythroid Progenitors Derived from Peripheral Blood

**Figure 1:**
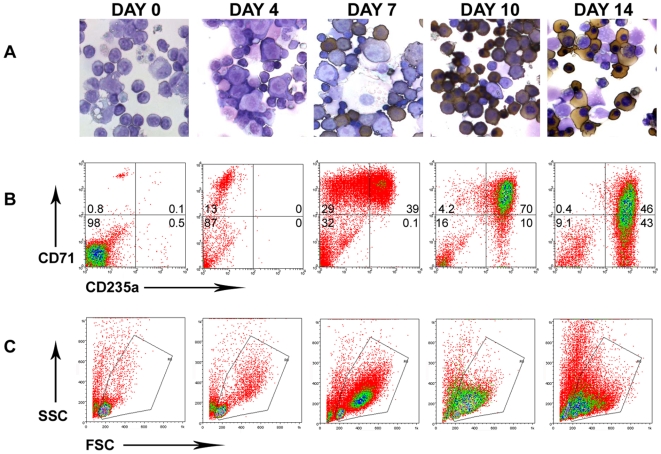
Erythroid Expansion *in vitro*. **A**, Cytospins of cells stained with 1% dianisidine/10% Giemsa are shown to illustrate the morphological changes within this culture as erythroblasts differentiate from larger basophilic cells on day 7 to smaller hemoglobin positive cells on day 10 and with pyknotic nuclei on day 14. The brown stain is indicative of hemoglobinization. **B**, The generation of erythroid precursors was monitored by labelling cells with fluorescent antibodies specific to the cell surface markers CD71 (transferrin receptor) and CD235a (glycophorin A) and analysed by flow cytometry. **C**, The changes in FSC and SSC of gated cells are shown in B to indicate the reduced size of erythroid precursors. Representative plots and cytospins viewed at ×60 magnification from 3 or more independent experiments are shown.

The well characterised two step liquid culture described by Fibach and colleagues [46] generates erythroblasts from peripheral blood mononuclear cells (PBMCs) and mimics the stages of transcription factor and globin expression that occur during adult erythropoiesis [47]. As a result each stage of erythroid development can be studied as shown in Figure 1. This system was used to examine the effect of malarial pigment on erythroid development.

The effect of malarial pigment on erythropoiesis was first studied by adding hemozoin to erythroid cultures on day 0 of the Epo dependent phase of culture (see Materials and Methods). Erythroid development was determined by assessing expression levels of transferrin receptor (CD71) and glycophorin A (CD235a) by flow cytometry.

**Figure 2:**
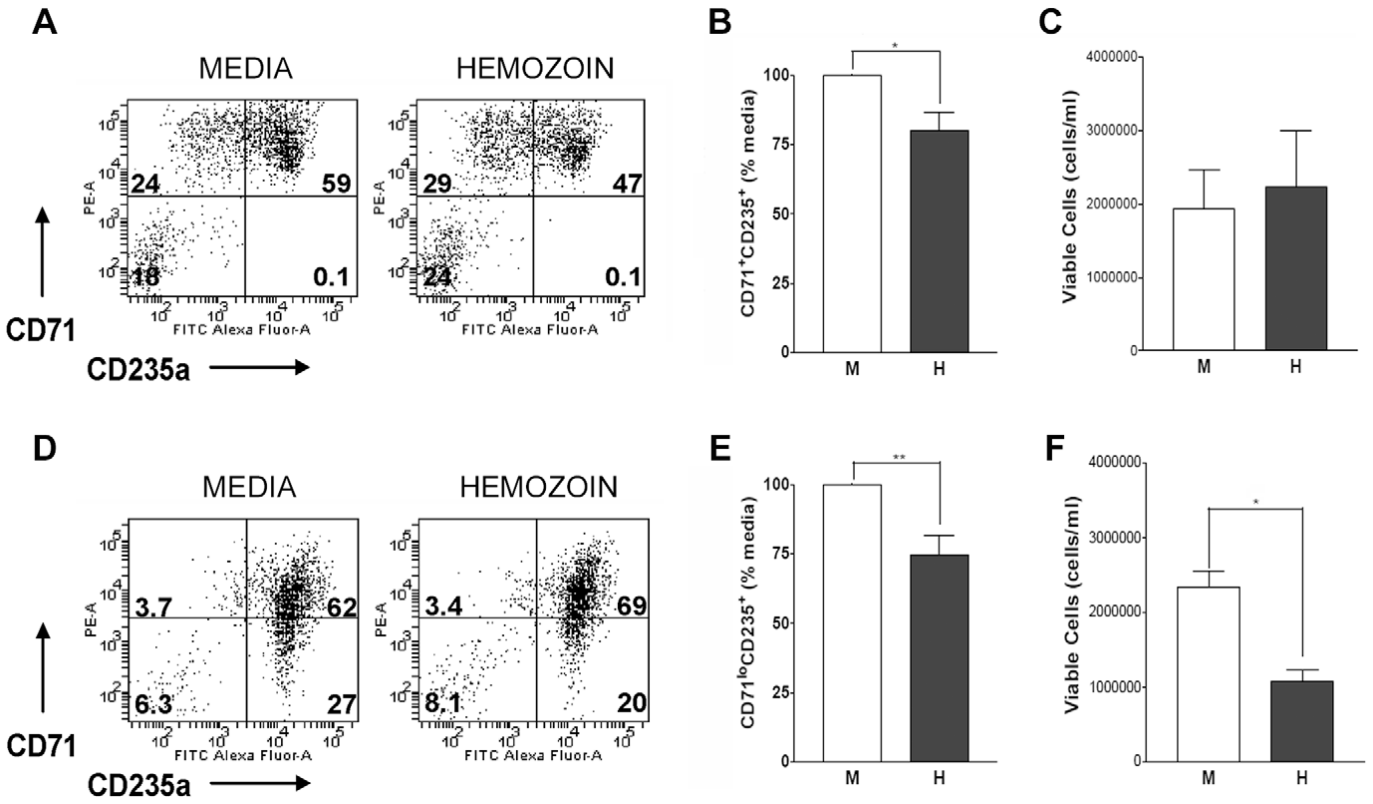
Hemozoin inhibits cell expansion and differentiation of erythroid progenitors. **A**, An example of the inhibition of progression to basophilic CD71^+^CD235^+^ precursors on day 7 is shown and **B**, the proportion of cells incubated with 6µg/ml hemozoin (H) that express CD71^+^CD235^+^ on day 7 is compared to media controls (M). * p = 0.030. **C**, The absolute number of viable cells on day 7 in control cultures with media or with hemozoin. **D**, An example of reduction in CD71^lo^CD235^+^ precursors on day 14 and **E**, the proportion of cells incubated with hemozoin that express CD71^lo^CD235^+^ on day 14 is compared to media controls. **p = 0.006. **F**, The absolute number of viable cells on day 14 following culture with hemozoin or control cultures. *p = 0.012. Dot plots show the percentage of live cells expressing markers for each quadrant. Bar graphs show the average of 3 or more independent experiments with SEMs where for each experiment values for CD71^+^CD235^+^ or CD71^lo^CD235^+^ have been normalized to cultures grown in media alone.

We found that hemozoin (equivalent to 6µg/ml heme equivalents) reduced the proportion of cells in culture expressing CD71 and CD235a on day 7 (Figures 2A and 2B). Examination of erythroid cultures on day 14 also showed that hemozoin reduced the proportion of more mature erythroid cells that express low levels of CD71 (Figures 2D and 2E). These observations suggested that some erythroid precursors had either died after exposure to hemozoin on day 0 of culture or that hemozoin may have delayed the maturation of erythroid precursors.

We therefore determined the number of surviving erythroid cells in culture by assessing the proportion of all cells expressing CD71 and/or CD235a and combining this value with the viable count of cells in culture on day 7 and day 14 (see Methods). Hemozoin added to cultures at 6µg/ml reduced the number of live erythroid progenitors recovered on day 14 but had no effect on the recovery of cells on day 7 compared with media alone (Figures 2C and 2F).

### Generation of Reactive Oxygen Species by Hemozoin

**Figure 3:**
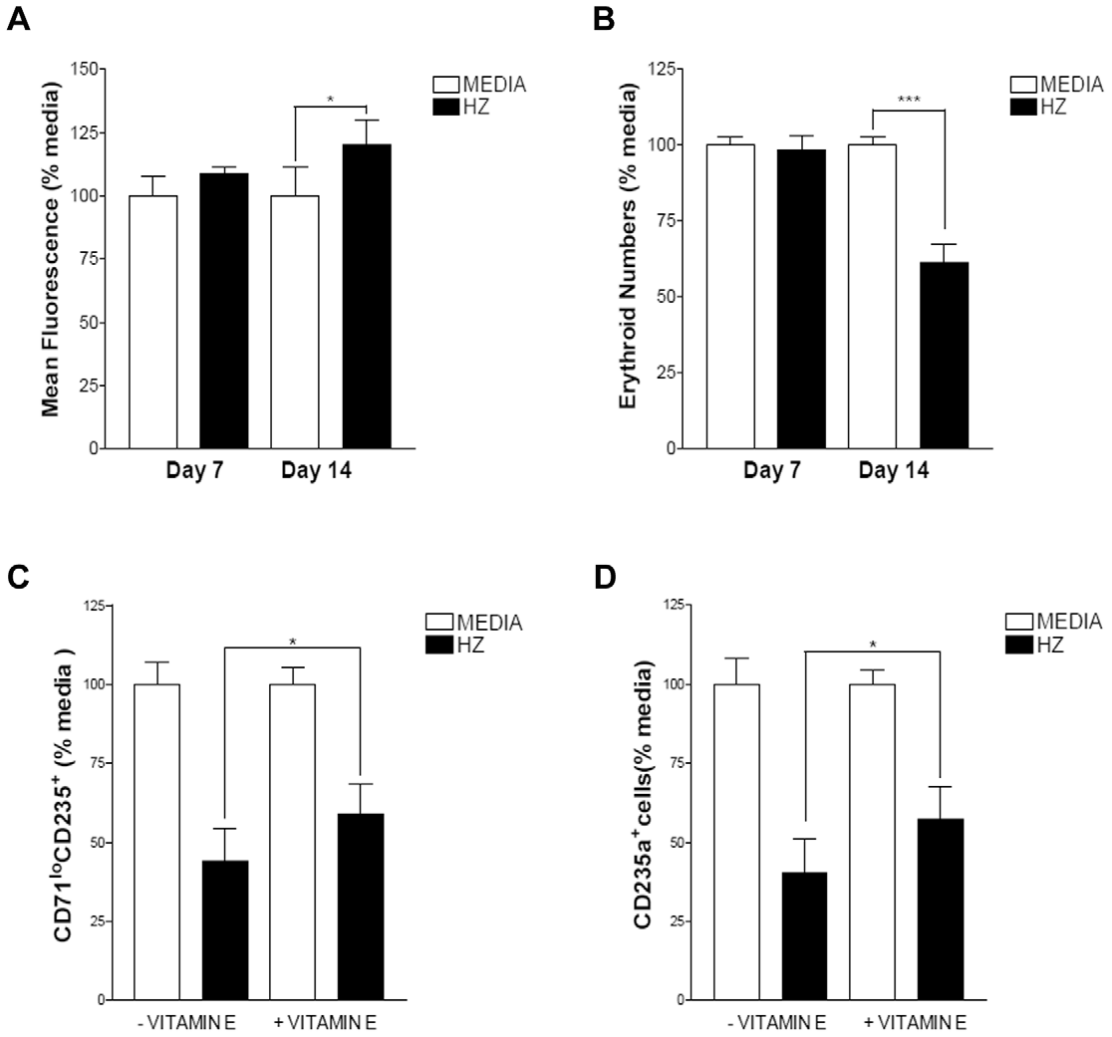
Assessment of reactive oxygen species (ROS) in erythroid cells following incubation with hemozoin. **A**, Relative levels of ROS were determined using the fluorescent dye 2, 7-dichlorofluoroscein diacetate. The mean fluorescence intensity relative to media controls at each time point is shown. **B**, Viable erythroid cells in the same cultures with controls (MEDIA) or with 6µg/ml hemozoin (HZ). **C**, The proportion of late erythroblasts (CD71^lo^CD235a^+^) and **D**, the total number of CD235a^+^ erythroid cells in culture on day 14 when treated with the anti-oxidant vitamin E. Cells were incubated with 6µg/ml hemozoin on day 0 or with the same dose of hemozoin after pre incubation with 30µM vitamin E and normalized to media controls in the absence or presence of vitamin E respectively. The averages of 3 independent experiments with SEMs are shown. **A** * p = 0.021, **B** *** p = 0.0003 **C** * p = 0.0278 and **D** * p = 0.0236.

The biological activity of hemozoin in previous studies has been attributed to its ability to induce oxidative stress. The generation of ROS in viable cells was therefore determined using the fluorogenic dye 2, 7-dichlorofluoroscein diacetate. After uptake by cells this dye is hydrolyzed by intracellular esterases and then oxidized by ROS to form dichlorofluoroscein which emits green fluorescence following excitation at between 480 and 500nm. We used flow cytometry to allow us to gate specifically on CD71^+^ cells (day 7) or CD235a^+^ cells (day 14). Hemozoin (6µg/ml) was added to erythroid cultures on day 0. Increases in ROS were observed on day 14 but not on day 7 (Figure 3A). Consistent with previous experiments there was a significant reduction in erythroid cells on day 14 that was absent on day 7 (Figure 3B).

The use of vitamin E succinate (succinyl ester of D-α-tocopherol), a potent cytoprotective and anti-oxidant reagent [48], [49] was used to reduce inhibition of erythroid cultures by hemozoin. Erythroid cultures from day 0 were pre-incubated with 30µM vitamin E succinate 1 hour before adding hemozoin. Vitamin E succinate reduced the inhibitory activity of 6µg/ml hemozoin on the maturation of precursors to CD71^lo^CD235a^+^ (Figure 3C) as well as the number of CD235a^+^ erythroid precursors in culture (Figure 3D).

### Modulation of Erythroid Development by Macrophages

**Figure 4:**
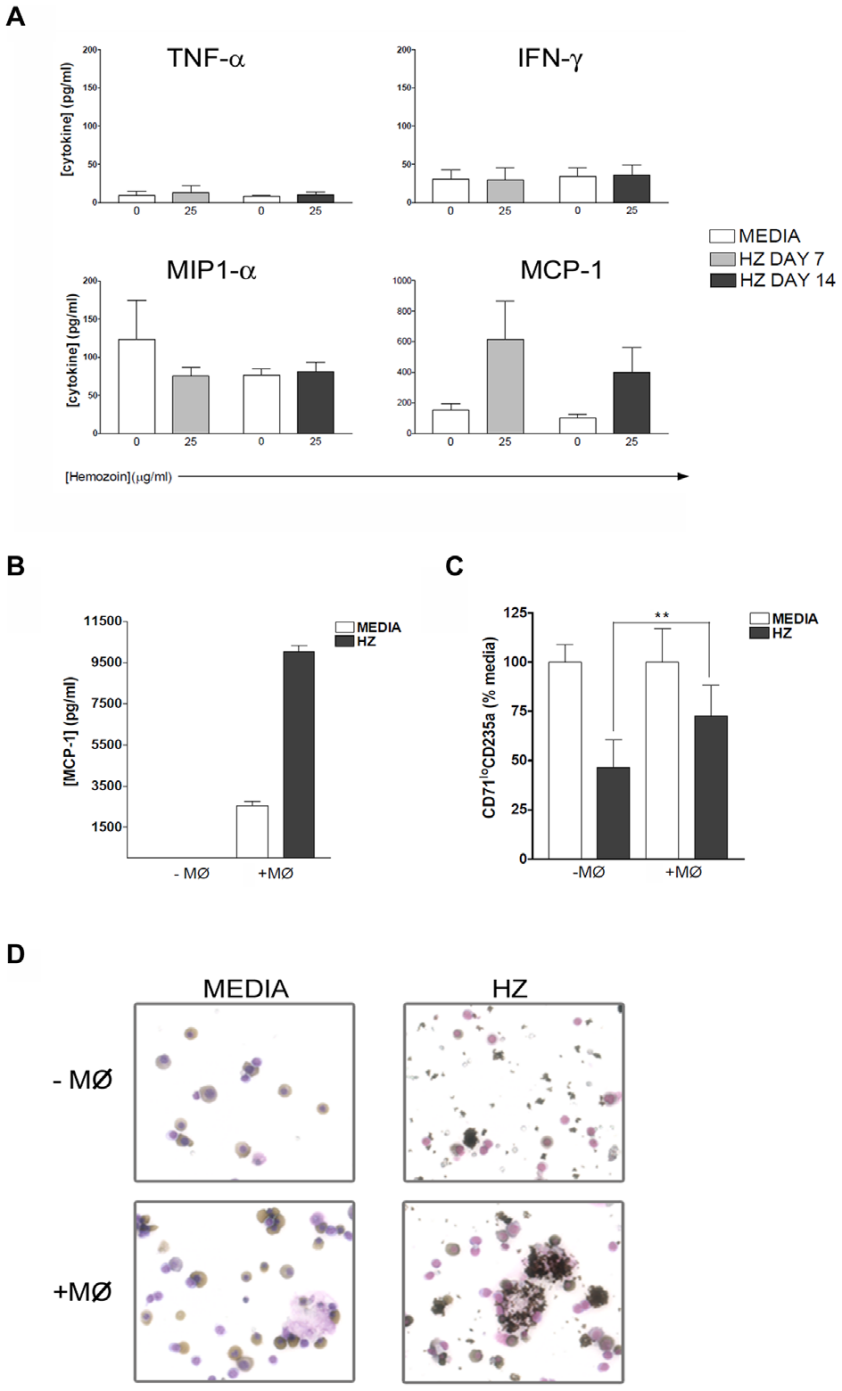
Macrophages do not contribute to erythroid inhibition induced by hemozoin. **A**, Cytokine levels in supernatants from erythroid cultures in the absence or presence of a high dose of hemozoin (25µg/ml) are shown for days 7 and 14. Empty bars represent levels detected in control cultures with media alone for each time point. The average of 3 or more independent experiments is shown with SEMs. **B**, Induction of MCP-1 by hemozoin is compared between erythroid cultures depleted of CD14^+^ macrophages on day 0 (−MØ) and cultures in which macrophages are present (+MØ). A representative experiment of 2 is shown with error bars as standard deviation of measurements in triplicate. **C**, The maturation of erythroblasts on day 14 to CD71^lo^CD235a^+^ precursors in cultures depleted of CD14^+^ on day 0 (−MØ) is reduced compared with the same cultures that have not had macrophages removed (+MØ). The effect of macrophage loss on erythroid development was taken into account by normalizing the yield of erythroblasts with hemozoin to the yield obtained in macrophage-depleted control cultures, where media but no hemozoin was added. The average of 2 independent experiments is shown where error bars are SEMs. **p = 0.003. **D**, O-dianisidine staining of cytospin preparations to show changes in morphology and hemoglobinization (brown staining) of cells cultured without macrophages and cultures with macrophages that are found associated with larger clusters of hemozoin. Images are representative of 3 or more experiments and were taken at ×40 magnification.

We wanted to establish whether macrophages, or cytokines secreted by them, mediated the inhibitory activity of hemozoin. Erythroblasts were cultured in the presence or absence of hemozoin and supernatants collected on days 7 and 14 were assayed for IFN-γ, IFN-α, TNF-α, MIP1-α and MCP-1. We were unable to detect IFN-α in any of the cultures studied (data not shown). There were detectable but low levels of other inflammatory mediators on day 7 and day 14 (Figure 4A). TNF-α (10–13 pg/ml) was 10 fold less than levels measured in patients with malarial anemia [12], [50], [51] and no increase in IFN-γ was observed upon addition of hemozoin. Only MCP-1 concentrations increased 4-fold to more than 400pg/ml on days 7 and 14 whilst the levels of MIP1-α were either slightly reduced or remained unchanged (Figure 4A). Furthermore, a blocking anti-TNF-α antibody added on day 0 of culture reversed the inhibition of exogenous TNF-α on erythroid growth but did not block the effect of hemozoin (Table 1).

**Table 1:** Neutralization of TNF-α does not reverse inhibition induced by hemozoin.

	InfliximAb	Erythroid Cells (mean±SD)	*p*-value (t-test)
Control	−	1666±420	
	+	1688±350	0.920
Hz	−	1295±427	
	+	1305±460	0.984
TNF-α	−	629±151	
	+	1547±365	0.087

Erythroid cultures were pre-incubated with 25µg/ml of the neutralising antibody to TNF-α, InfliximAb (Centocor, Horsham, USA) on day 0 of the erythropoietin dependent stage of culture. After 1 hour hemozoin (Hz) and TNF-α were added at 6µg/ml and 10ng/ml respectively. The number of live erythroid CD235a positive events acquired (out of a total of 10, 000) by flow cytometry on day 14 is shown. Data from a representative experiment of 3 is shown.

Erythroid cultures were next depleted of macrophages (CD14^+^ cells) on day 0. Flow cytometry analysis indicated that CD14^+^ macrophages typically constituted 1–3% of total cells analyzed and were reduced more than 10-fold after depletion (data not shown). As described previously [52] the yield and differentiation of erythroid cells is reduced in the absence of macrophages. To control for this effect, we assessed erythroid maturation in cultures depleted of macrophages both with and without hemozoin.

When supernatants from these cultures were assayed for MCP-1 we found that its induction by hemozoin required the presence of CD14^+^ cells since their removal resulted in no induction of MCP-1 by hemozoin (Figure 4B). Removal of macrophages also increased the inhibitory effect of hemozoin. We found that fewer CD71^lo^ CD235a^+^ cells survived in culture in the presence of hemozoin when macrophages were absent (Figure 4C). This was also observed with β hematin, a synthetic form of hemozoin (data not shown). It was therefore apparent that the inhibitory effect of hemozoin was less in the presence of MCP-1 and it seemed likely that the role of MCP-1 in this system is to modulate monocyte function [53].

We also found that cultures depleted of CD14^+^ cells had smaller aggregates of pigment dispersed amongst erythroid cells (Figure 4D, -MØ). In contrast, cultures with CD14^+^ cells often had macrophages in association with larger aggregates of pigment indicating phagocytosis of hemozoin (Figure 4D, +MØ).

**Figure 5:**
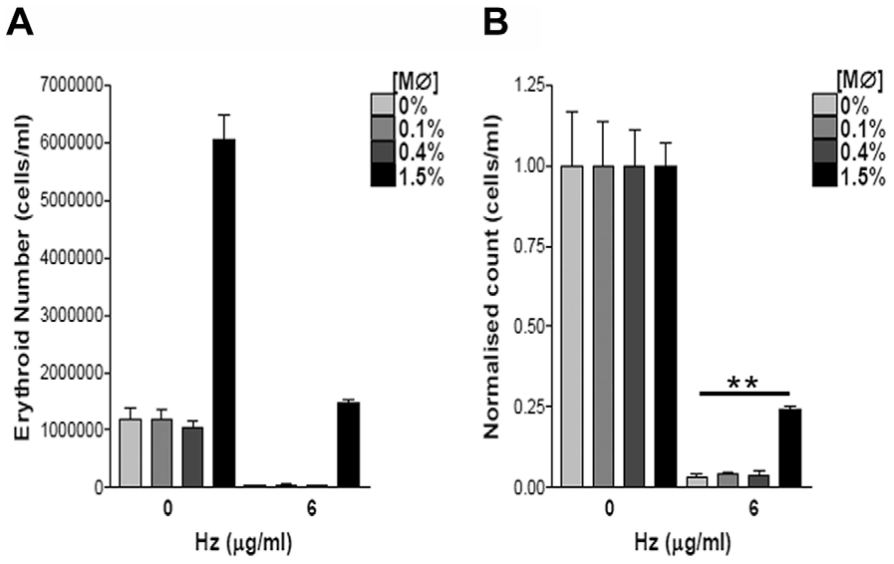
Macrophage content required to reduce inhibition by hemozoin. Increasing proportions of isogenic CD14^+^ cells (MØ) isolated from the same culture were added on day 0 together with hemozoin. **A**, Absolute numbers of erythroblasts on day 14 and **B**, normalization of the same cell counts to media controls with each concentration of CD14^+^ cells to allow assessment of rescue by CD14^+^ cells. A representative experiment of 3 is shown where error bars are SDs of measurements in triplicate. ** p = 0.007.

The content of CD14+ cells in cultures was varied to determine the minimal proportion of macrophages required to reduce inhibition by hemozoin. To avoid cytotoxic activity due to non-matched cells the CD14-negative cultures were generated from the same donor as the non-separated cells. On day 14 the absolute number of more mature erythroblasts was determined (Figure 5A). After normalization to media controls containing the same proportion of CD14+ cells, an enhancement of cells grown with hemozoin was only observed with 1.5% CD14^+^ where erythroid numbers were 25% of those seen in control cultures with the same proportion of macrophages (Figure 5B).

These results demonstrated the potential role of macrophages in supporting erythropoiesis during malaria infection by reducing contact between hemozoin and erythroid cells. Furthermore the inhibitory effects of hemozoin were not dependent on release of pro-inflammatory mediators from the macrophage.

### Apoptotic Pathways Induced by Hemozoin in Erythroid Precursors

**Figure 6:**
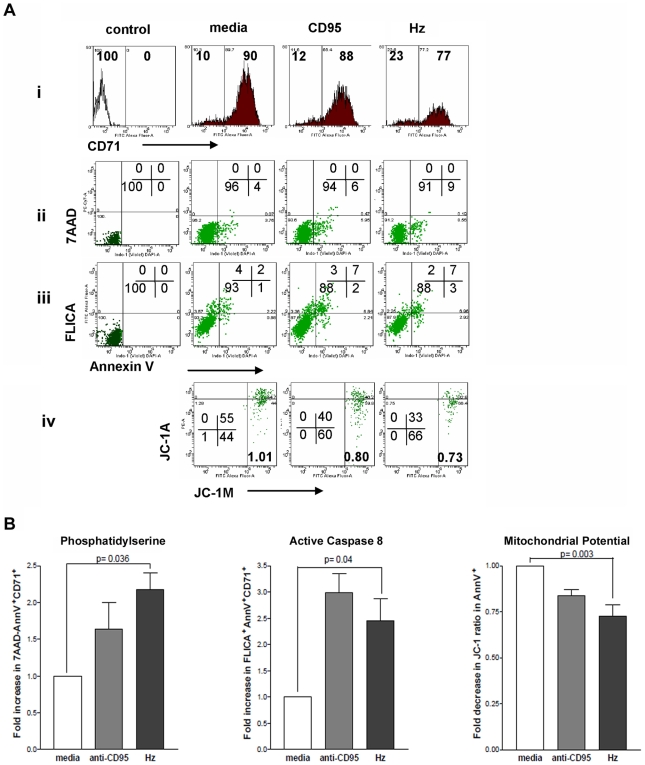
Apoptosis of erythroid cells is enhanced by hemozoin. **A**, Basophilic erythroblasts from day 6 cultures were incubated with 20µg/ml anti-CD95 (CD95) or hemozoin at 6µg/ml (Hz) and 24 hours later assessed for **i**, the relative proportion of CD71^+^ erythroblasts **ii**, exposure of phosphatidylserine on non necrotic 7AAD^−^ cells and **iii**, activation of caspase 8 (FLICA LETD^+^) in Annexin V^+^ cells; **iv**, a proportion of cells from the same experimental groups were also assessed for permeability of mitochondria using the fluorescent dye JC-1. Fluorescence from JC-1 aggregates in mitochondria (JC1-A) and from monomers in the cytosol (JC1-M) is used to calculate the ratio of JC1-A to JC1-M to allow for comparison between experiments. Loss of membrane potential is indicated by values less than 1 shown in bold in the lower right quadrant of each dot plot. The proportion of cells with JC1-A^hi^ and JC1-A^lo^ fluorescence is also shown to the left. Controls are isotype controls. **B**, The average of 3 or more independent experiments as described in A following incubation with media, anti-CD95, or hemozoin (Hz) demonstrating fold changes in apoptotic markers. Error bars are of SEMs and p values are from analyses using the student's t test.

Since hemozoin appeared to have a significant effect on the survival of erythroid precursors as well as on their maturation we determined the role of hemozoin in the induction of apoptosis in erythroid cells. To investigate which apoptotic pathway is involved, we studied the effects of hemozoin on the basophilic erythroblast population appearing on days 6 to 7 of culture. Day 6 erythroid cultures were incubated with hemozoin at 6µg/ml and cells harvested to assess levels of pro-apoptotic markers. Within 24 hours we observed loss of CD71^+^ erythroid precursors (Figure 6Ai) accompanied by elevated levels of exposed phosphatidylserine (Figure 6Aii and 6B). Within the same population of cells we also observed an increase in activated Caspase 8 by staining with a fluoresceinated inhibitor of caspase 8 (FLICA-LETD) (Figures 6Aiii and 6B). When a proportion of the same cells exposed to hemozoin were incubated with the mitochondrial cationic dye, JC-1, increased disruption of the mitochondrial membrane potential in Annexin V^+^ cells was also observed (Figures 6Aiv and 6B). We used 20µg/ml of an antibody that cross links the death receptor, CD95, as a positive control and found that the proportion of cells with activated caspase 8 and decreased mitochondrial membrane potential was comparable to that seen with 6µg/ml hemozoin (p>0.1 with students t test).

Reduction in mitochondrial membrane potential is consistent with disturbance of membrane function through either the generation of ROS or activation of Bid by cleaved Caspase 8. Activated Bid (tBid) mediates mobilization of Bax to mitochondria which results in membrane permeabilization and release of Cytochrome C. Released Cytochrome C forms an apoptosome with Apaf I to cleave and activate Caspase 9 and also is an inducer of active Caspase 3 [54].

**Figure 7:**
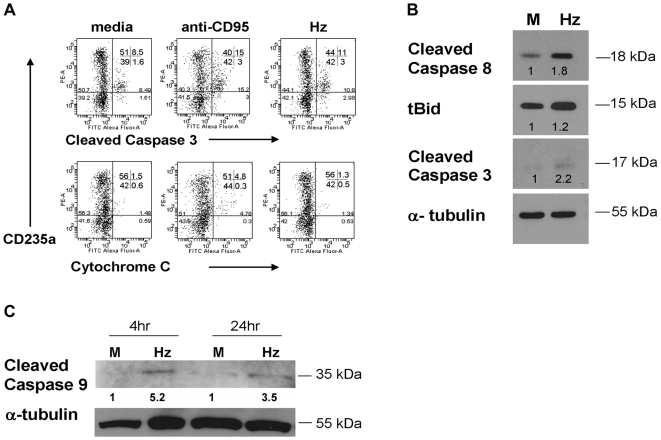
Activation of Caspases in erythroid cells. **A**, Induction of cleaved caspase 3 and cytochrome C in basophilic erythroblasts purified from day 6 cultures 24 hours after incubation with media (controls), anti-CD95 or 6µg/ml hemozoin. **B**, Western blots of lysates taken from purified basophilic erythroblasts incubated with hemozoin for 4 hours demonstrate increased levels of cleaved caspase 8 and activation of caspase 3 with slight induction of cleaved Bid (tBid). **C**, Detection of cleaved caspase 9 after 4 and 24 hours incubation with hemozoin. Each band was normalised to that for alpha tubulin. Fold changes in normalized levels of pro-apoptotic proteins relative to those in lysates from media controls are shown below each band.

We therefore also determined whether we could detect increased levels of activated caspase 3 and cytochrome C in the cytosol of cells incubated with hemozoin. Erythroblasts were purified from erythroid cultures on day 6 and then incubated with 6µg/ml hemozoin for 24 hours. Using flow cytometry we observed measurable but low increases in activated caspase 3 but not of cytochrome C (Figure 7A). Western blots were also performed on lysates from purified erythroblasts incubated with the same dose of hemozoin for 4 hours (Figure 7B). Here we observed elevated levels of cleaved Caspase 8 and cleaved Caspase 3 and comparatively lower elevation of tBid (Figure 7B). However cytosolic cytochrome C was not induced in cultures incubated with hemozoin (data not shown). The same lysates contained elevated levels of cleaved Caspase 9 after 4 and 24 hours (Figure 7C) where the levels of cleaved Caspase 9 were increased up to 5-fold following normalization to alpha tubulin. Taken together these results indicated that hemozoin can increase levels of cleaved (activated) caspase 8 and activated tBid and that this corresponded with disruption of the mitochondrial membrane potential and increases in cleaved (activated) caspases 3 and 9 in erythroid precursors.

## Discussion

Inadequate production of reticulocytes from the bone marrow in response to the removal of circulating erythrocytes is a significant component of the pathology of severe anemia in falciparum malaria infection. The characteristic features of severe malarial anemia (SMA) include hemoglobin levels of 5g/dL or less, increased clearance of infected and uninfected erythrocytes, inadequate reticulocyte responses and dyserythropoiesis of the bone marrow with phagocytosed erythroid precursors within macrophages [3], [24], [55]. Here, we show for the first time that macrophages can protect erythroblasts from the inhibitory effects of hemozoin and also show that inhibition of erythroid expansion is accompanied by apoptosis of erythroid precursors.

We found that, *in vitro*, hemozoin had a greater effect on the numbers of day 14 mature orthochromatophilic erythroblasts (CD71^lo^CD235a^+^) compared to the younger basophilic cells (CD71^+^CD235a^+^) on day 7. Furthermore, there was increased hemozoin-induced exposure of phosphatidylserine on CD71^hi^ erythroid cells. If the proportion of apoptotic cells is taken into account then the effect of hemozoin on the production of fully functional erythroblasts is considerably greater than suggested by cell counts alone.

We have shown here that hemozoin enhanced levels of cleaved caspase 8 and caspase 3 and also disturbed the membrane potential of mitochondria. However there was no evidence that pro-inflammatory cytokines were induced by hemozoin or that they contributed to the enhanced apoptosis in our erythroid cultures derived from PBMCs. These results are consistent with previous observations using erythroid precursors from CD34+ cells where hemozoin (and its synthetic analogue β hematin) inhibited erythroid growth and development independent of TNF-α and IFN-γ [4]. Suppression of the reticulocyte response was associated with levels of hemozoin circulating in plasma or in phagocytic cells where the concentrations of hemozoin found in plasma were comparable to those used in the study described here.

We found that after adding hemozoin, macrophages are associated with pigment and that removal of CD14^+^ monocytes or macrophages from culture decreased the growth of erythroid cells and increased the inhibitory effect of hemozoin on erythropoiesis. It is possible that a component of protection was mediated via the growth enhancing effects of macrophages [56] as well as via the removal of malarial pigment by macrophages. However, it is clear that macrophages added to developing erythroid cells in the presence of hemozoin do not increase the inhibitory effect of hemozoin on erythroid development. We were unable to completely overcome the inhibitory effects of hemozoin with an excess of CD14^+^ cells, probably due to a combination of the limited numbers of isogenic CD14^+^ cells recovered in each experiment and the previously reported inhibitory effects of hemozoin on macrophage function [25].

Monocytes and macrophages have multiple roles in malaria infection including specific roles in removal of iRBCs by phagocytosis of mature and ring-stage forms [57], [58], antibody-dependent cytotoxicity [59] and more generally by stimulation of the innate immune response during the early stages of infection (for review see reference [23]). In children admitted to hospital with malaria, a low circulating monocyte count is associated with a poor outcome, independently of the other clinical features or other leukocyte counts [60]. Our data showing reduced inhibitory effect of hemozoin on erythroid precursors when macrophage numbers are increased would be consistent with these clinical observations and add to the roles of these cells during malaria infection.

In malaria endemic areas of Africa up to 6–8% of circulating white blood cells in children are monocytes [60], [61]. It is conceivable that with physiological levels of functional non-pigmented monocytes in circulation the inhibitory effects of hemozoin could be completely overcome. There are few studies that provide differential counts from the bone marrows of children with malaria. However increases in circulating monocytes containing pigment are indicative of long protracted or repeated infections in which severe malarial anemia is more likely than cerebral anemia and is an indicator of overall sequestered parasite burden [62]. Very early studies report that repeated attacks of malaria can result in grey discoloration of the bone marrow due to accumulation of hemozoin (reviewed in [5], [63]). In such situations macrophage dysfunction secondary to phagocytosis of hemozoin might limit further ingestion of hemozoin and/or infected red blood cells [25]. Our observations indicate that failure of phagocytic cells to clear hemozoin may severely reduce the erythropoietic response to hemolysis of RBCs during infection.

A direct effect of malarial pigment on erythroid development would be to increase the level of oxidative stress in these cells. Low levels of vitamin E in the RBC membranes of children with malaria have been associated with the severity of malarial anemia [64]. Although the source of ROS in this study was not identified it is possible that low levels of α-tocopherol may be associated with reduced survival of developing erythroblasts in contact with hemozoin. We were able to detect significant increases of ROS in developing precursors with 6µg/ml hemozoin and pre-incubation with an anti-oxidant reduced the inhibitory effects of hemozoin.

Apoptosis is involved in the homeostatic mechanisms for erythropoiesis and can occur via intrinsic activation of caspases, withdrawal of Epo or pro-inflammatory stimulation of death receptors. In our cultures, erythropoietin levels (1000mU/ml) are comparable with those seen in children with SMA and so do not limit erythropoiesis. Inflammatory cytokines are also absent, suggesting that hemozoin may directly or indirectly stimulate apoptosis by an intrinsic route and/or by increased expression of death receptors or their ligands.

It is possible that hemozoin in contact with cells in the bone marrow mediates ineffective erythropoiesis by inducing apoptosis of erythroblasts. We found that hemozoin had a direct impact on the extrinsic apoptotic pathways of basophilic erythroid precursors. Hemozoin was observed in contact with erythroid cells during their culture and EM studies would confirm whether hemozoin is also within erythroblasts. Our experiments have used only a single exposure to hemozoin to demonstrate inhibition of erythropoiesis. It is possible that repeated release of hemozoin at each cycle of parasite growth *in vivo* may have a cumulative and greater effect on erythroid development than suggested by these *in vitro* experiments. Ineffective erythropoiesis as a result of enhanced apoptosis is also observed in Thai thalassaemia patients due to an excess of α-globin [65].

Previous studies have shown that expression of caspase 3 is transiently elevated during the proerythroblast and basophilic stages of development [66]. In the cultures described here caspase 8 was also expressed at this stage of differentiation. After only 4 hours of incubation with hemozoin, the activated forms of both these enzymes were increased compared to controls. Elevation of cleaved caspase 8 indicated that some of the observed loss of mitochondrial potential stems from the extrinsic pathway which may in turn be amplified through activation of caspase 9.

Of note is the inability to detect cytochrome C with hemozoin despite the observed disruption of mitochondria equivalent to that observed after cross linking the death receptor CD95. Since we detected the cleaved product of caspase 9 both at 4 and 24 hours this suggests that either hemozoin induced an undetectable but sufficient quantity of cytochrome C to cause some activation of caspase 9 or that activation occurred by an alternative route. These observations are supported by a previous report that describes an 80% drop in cytochrome C and only transient caspase 3 and 9 activity in cultured erythroid cells as they mature [67].

We have not been able to examine the role of other stromal cells and we cannot exclude that they or other accessory cells may participate in pathology or protection during malaria. Similarly, our observations do not rule out the contribution of systemic mediators of inflammation to dyserythropoiesis observed during acute malaria infection *in vivo*. Several studies investigating the role of apoptosis in the pathology of severe malaria indicate that contact of parasitized RBCs or neutrophils with endothelial cells [68], [69] can induce apoptosis of vascular endothelia and neuroglia, whilst others suggest a role of soluble factors from parasitized cells [70], [71].

In our studies we examined the role of macrophages in modulating the inhibition induced by hemozoin on erythroid development. Our observations are consistent with a direct effect of hemozoin on erythroid precursors. Hemozoin increased levels of ROS and apoptosis in erythroblasts. Activation of caspase 8, mitochondrial disruption, caspase 9 and 3 were observed implicating both the extrinsic and intrinsic pathways of apoptosis. Further work is required to elucidate the multiple pathways of cellular dysfunction induced by hemozoin and how this disrupts the ‘erythroblast survival network’ [72]. Since the data indicate that hemozoin can act directly on erythroid precursors to inhibit normal erythropoietic activity, these observations may be relevant to the effect of hemozoin on other host cells. Further studies will be required to determine the molecular basis of this inhibition and to correlate these observations with those in individuals with severe malaria.

## Materials and Methods

### Materials

All tissue culture grade reagents were obtained from (Sigma, Poole, UK) unless otherwise stated.

### Tissue Culture

The 3D7 strain of *P. falciparum* was up to 10% parasitemia at 1–2% hematocrit as described previously [73], [74]. Erythroid cultures were grown from PBMCs in a two-stage system [46]. Briefly, PBMCs were isolated from a buffy coat (NHSBT, Bristol, UK) and early erythroblasts were expanded for one week in 10% conditioned medium from the bladder cancer cell line 5637, 10% non-heat inactivated FBS (SLI, Salisbury, UK) and 1µg/ml Cyclosporin A (Sandoz Pharmaceuticals, Surrey, UK) in α-modified MEM. Erythroid cells were washed twice before transfer into α-MEM with 1000mU/ml recombinant Epo (Ortho Biotech Janssen Cilag Ltd, Bucks, UK), 10ng/ml SCF (R&D Systems, Abingdon, UK), 0.3 mg/ml holo transferrin (MP Biomedical, London, UK), 1µM dexamethasone (Faulding Pharmaceuticals plc, Warwickshire, UK), 3% non-heat inactivated FBS (SLI) and 1% fraction V deionized BSA. The start of this second stage of culture is referred to as day 0. Erythroid precursors differentiated into mature erythroblasts and hemoglobinized normoblasts over the next 14 days. The proportion of erythroid cells was typically 60–70% on day 7 and 80% on day 14 of the second phase of culture. Non-erythroid cells on day 7 of the second phase consisted of CD14+ cells (1–3%), CD20+ cells (5%) and CD3+ T cells (22%). These diminished proportionally as erythroid cells expanded. Experiments were performed on cells from the second stage seeded in 0.5ml at 5×10^5^ cells per well of a 48 well dish. For experiments studying the influence of macrophages on the activity of hemozoin, CD14^+^ cells were isolated at the end of the first phase of culture using the CD14 microbead isolation kit (Miltenyi Biotec, Guildford, UK) and were incubated in the second phase with remaining cells from the same buffy coat as indicated.

### Isolation and Preparation of Malarial Pigment

Mycoplasma-free *P. falciparum* (3D7) cultures were enriched for trophozoites and schizonts over 60% Percoll [75] and lysed in 8µg/ml digitonin for 10 minutes on ice. Centrifugation at 16400g for 10 minutes at 4°C was followed by sterile sonication (Soni prep 150, MSE) in 2% SDS Tris pH8 for 2s at amplitude of 10 microns on ice. Hemozoin was obtained after 4–5 washes in 2% SDS Tris pH8, one wash in 1% Triton X-100 and finally 3 washes in 100-fold volumes of PBS, before storage under nitrogen at −80°C. Before use all pigment preparations were sonicated for 10–20s as described above.

### Quantification of Pigment

The concentrations of hemozoin were determined following depolymerization in 20mM NaOH for 2 hours at room temperature [76]. The absorbance at 405nm was compared with known concentrations of α-hematin and the concentration determined as hematin equivalents/ml. The quantity of infected RBC required to produce a given amount of hematin equivalents per ml was calculated assuming 1×10^7^ RBCs/µl.

### Flow Cytometry

Erythroid cell maturation was determined by measuring expression of CD71 and CD235a with the appropriate mAbs (Beckman Coulter plc, High Wycombe, UK and DAKO Cytomation Ltd, Ely, UK). Monocytes and macrophages were identified with murine anti-CD14 (Serotec, Oxford, UK), using appropriate isotype controls. Cells were stained in 0.5% BSA, 0.05% sodium azide in PBS for 30 minutes at 4°C, washed twice and fixed in 2% paraformaldehyde. Live cells were identified from FSC and SSC profiles of 7-AAD (Becton Dickinson, Oxford, UK) -negative unfixed cells. The absolute number of erythroid cells was determined by multiplying the viable cell count by the proportion of CD71^+^ and/or CD235a^+^ cells acquired. Countbright fluorescent beads (Invitrogen, Paisley, UK) were also used to determine erythroid numbers in culture. For the detection of early apoptotic events, cells were stained with PE conjugated anti-CD71, washed twice with Annexin V binding buffer and incubated with fluoresceinated Annexin V (R&D Systems, Abingdon, UK). Cells were analysed within 1 hour following the addition of 7-AAD to permit exclusion of dead cells. To detect cleaved caspases and cytochrome C, cells were stained for extracellular markers, fixed in 2% paraformaldehyde for 10 minutes at 37°C, chilled on ice for 1 minute. After removal of paraformaldehyde, cells were permeabilised with ice cold absolute methanol, incubated on ice for 30 minutes, washed twice with PBS containing 0.5% BSA and stained with antibodies to either cleaved caspase 3 or cytochrome C (Cell Signaling Technology, Hitchin, UK). Cells were washed and analyzed on the same day by flow cytometry on a FACSCalibur or LSR II machine (Becton Dickinson) at a flow rate of <1500 events/s. Events were analysed with WinMDI (http://facs.scripps.edu/software.html) and Weasel software (http://www.wehi.edu.au/faculty/advanced_research_technologies/flow_cytometry/weasel_for_flow_cytometry_data_analysis/).

### Identification of Apoptotic Markers

Day 6 cells from the second stage of culture were incubated with hemozoin or 20µg/ml anti-CD95 (clone DX2, Becton Dickenson) cross-linked using 11µg/ml protein G. The levels of activated caspase 8, the mitochondrial potential and exposed phosphatidyl-serine were determined using the FLICA kit, the JC-1 kit and Alexa 350-Annexin V respectively (Invitrogen). Loss of membrane potential was determined as MFI of aggregates (JC1-A)/MFI of monomers (JC-1M). Calculating the ratio of fluorescence due to each form of JC-1 reduces the influence of different mitochondrial size, shape or density when quantifying fluorescence due to released monomers. This therefore allows comparative measurements to be made between experiments. Cells were stained for FLICA and JC-1 following the manufacturer's instructions and for CD71, Annexin V and 7-AAD as described previously. For Western blot analyses day 6 cells were enriched for erythroid progenitors using magnetic bead isolations (Miltenyi Biotec). Briefly CD3^+^ cells were depleted with an LD column and CD71^+^ cells selected from the excluded fraction using an LS column. Eluted cells from the final step were incubated with hemozoin and pelleted at 4°C for 10 minutes at 400g, washed with PBS containing a protease inhibitor cocktail, resuspended in 50µl of cell lysis buffer containing 20mM TrisHCl pH7.5, 2mM EDTA, 150mM NaCl, 0.5% Triton X- 100, 1mM PMSF, inhibitor cocktails to proteases (containing 4-(2-aminoethyl)benzenesulfonyl fluoride, E-64, bestatin, leupeptin, aprotinin, and sodium EDTA) and phosphatases (containing microcystin LR, cantharidin, (−)-p-bromotetramisole, microcystin LR, cantharidin, and (−)-p-bromotetramisole) and incubated on ice for 20 minutes. The crude lysate was homogenized by passing through a 25 gauge needle 30 times and cytosolic fractions collected after pelleting nuclei and mitochondria at 16,000g and 4°C for 15 minutes. Approximately 20µg protein per lane was loaded onto 12% Tris-glycine polyacrylamide gels (Bio-Rad Laboratories, Hemel Hampstead, UK) and transferred onto Polyvinylidene Difluoride membranes using the iBlot system (Invitrogen). Membranes were probed with antibodies to cleaved forms of caspase 3, caspase 8 and Bid (Cell Signaling Technology) and proteins visualized with Dura Sensitive Rapid detection kit (Pierce Biotechnology, Cramlington, UK).

### Microscopy

Cytospins were performed by loading 1−2×10^5^ cells in 150µl into Shandon cytospin funnels (Thermo Electron Corporation, Massachusetts, USA) and centrifuged for 3 minutes at 1000rpm. Cells were fixed in absolute methanol and hemoglobinized cells identified using 1% (w/v) O-dianisidine in MeOH for 5 minutes. The reaction was stopped using 2.5% H_2_O_2_ in 70% EtOH for 2–3 minutes and cells counterstained using 10% (w/v) Giemsa in water (pH6.8) before rinsing with water.

### Cytokine ELISAs

Tissue culture supernatants were collected and stored at −20°C. Samples were thawed on ice and levels of TNF-α, IFN-α, IFN-γ, MIP-1α and MCP-1 were determined using Quantikine kits (R&D Systems). Cytokine concentrations were calculated from duplicate readings with reference to standards in the kit.

### Statistical Analyses

Unless stated differently statistical significance was determined with GraphPad Prism using the two-tailed paired student's test at 95% confidence interval.
